# Use of human fibrin glue (*Ti*sseel) versus *sta*ples for mesh fixation in laparoscopic transabdominal preperitoneal hernioplasty (TISTA): a randomized controlled trial (NCT01641718)

**DOI:** 10.1186/1471-2482-14-18

**Published:** 2014-04-01

**Authors:** Sascha A Müller, Rene Warschkow, Ulrich Beutner, Cornelia Lüthi, Kristjan Ukegjini, Bruno M Schmied, Ignazio Tarantino

**Affiliations:** 1 Department of Surgery, Kantonsspital St.Gallen, CH-9007 St. Gallen, Switzerland; 2 Institute of Medical Biometry and Informatics, University of Heidelberg, D-69120 Heidelberg, Germany; 3 Department of Surgery, Heidelberg University Hospital, D-69120 Heidelberg, Germany

**Keywords:** Hernia repair, Fibrin glue, Staples, Mesh, Laparoscopy, Postoperative pain

## Abstract

**Background:**

Inguinal hernia repair is one of the most common surgical procedures worldwide. This procedure is increasingly performed with endoscopic techniques (laparoscopy). Many surgeons prefer to cover the hernia gap with a mesh to prevent recurrence. The mesh must be fixed tightly, but without tension. During laparoscopic surgery, the mesh is generally fixed with staples or tissue glue. However, staples often cause pain at the staple sites, and they can cause scarring of the abdominal wall, which can lead to chronic pain. We designed a trial that aims to determine whether mesh fixation with glue might cause less postoperative pain than fixation with staples during a transabdominal preperitoneal patch plastic repair.

**Methods/Design:**

The TISTA trial is a prospective, randomized, controlled, single-center trial with a two-by-two parallel design. All patients and outcome-assessors will be blinded to treatment allocations. For eligibility, patients must be male, ≥18 years old, and scheduled for laparoscopic repair of a primary inguinal hernia. One group comprises patients with a unilateral inguinal hernia that will be randomized to receive mesh fixation with either tissue glue or staples. The second group comprises patients with bilateral inguinal hernias. They will be randomized to receive mesh fixation with tissue glue either on the right or the left side and with staples on the other side. The primary endpoint will be pain under physical stress, measured at 24 h after surgery. Pain will be rated by the patient based on a numeric rating scale from 0 to 10, where 10 equals the worst pain imaginable. A total of 82 patients will be recruited (58 patients with unilateral inguinal hernias and 24 patients with bilateral hernias). This number is estimated to provide 90% power for detecting a pain reduction of one point on a numeric rating scale, with a standard deviation of one.

**Discussion:**

Patients with bilateral hernias will receive two meshes, one fixed with glue, and the other fixed with staples. This design will eliminate the inter-individual bias inherent in comparing pain measurements between two groups of patients.

**Trial registration:**

ClinicalTrials.gov: NCT01641718

## Background

### Scientific background and explanation of rationale

Inguinal hernias are the most common hernia; they account for 90% of all spontaneous hernias. Moreover, inguinal hernia repair is the most frequently performed procedure in general surgery. In Germany, more than 200,000 inguinal hernias are repaired annually [1,2]. The standard method for repairing an inguinal hernia, originally described by Bassini in 1889, is to close the inguinal canal with sutures. Due to the high recurrence rate with this technique [3], new methods were established that used tension-free implantation of synthetic meshes. Furthermore, endoscopic/laparoscopic methods have been introduced, where the hernia canal is typically approached from the posterior side; this approach is directly opposite to the anterior approach used in the open surgery technique. Which of these approaches and methods yields the better results is strongly debated [4]. Among the various posterior techniques employing preperitoneal mesh implantation [2,5-7], the two most widely accepted techniques are the transabdominal preperitoneal patch plastic (TAPP) repair and the total extraperitoneal (TEP) repair [8,9]. There are many indications for both techniques, but the TAPP repair is particularly recommended for recurrent hernias (after an open preperitoneal patch plastic) and difficult hernias (sliding or incarcerated hernias) [2,8,10]. The TAPP repair has the advantage that it is easier to perform, can be better standardized, and offers the possibility to perform a diagnostic laparoscopy [2]. Thus, the type of hernia can be assessed immediately on both sides of the groin, and a bilateral repair can be performed without additional incisions. In general, the TAPP repair is easier to learn than the TEP repair [2,10,11]. Most randomized studies that compared laparoscopic with open repair found that laparoscopy was associated with less postoperative pain, earlier return to work, higher costs, a longer operating time, a longer learning period, and a higher recurrence and complication rate during the early learning phase [12-14]. In summary, open, mesh-based, tension-free repair remains the standard procedure; however, in the hands of a well-trained surgeon laparoscopic herniorrhaphy can produce excellent results comparable with those of open repair [14,15].

Many factors determine whether patients experience postoperative pain, including the type of intervention, the presence of complications, patient age, and individual tolerance. In particular, the type of mesh fixation employed for the hernioplasty has a strong influence on postoperative pain [16,17]. In a recent review, mesh fixation with glue was compared to mesh fixation with staples for an endoscopic inguinal hernia repair [18]. For the TAPP repair, two randomized controlled trials [19,20], one non-randomized trial [21], and two case series [22,23] were reviewed. The authors of the review found less postoperative pain and more rapid recovery after glue fixation than after staple fixation, without any significant difference in the recurrence rate. Nevertheless, the authors concluded that, due to the overall poor quality of the currently published data, future well-designed studies are required to demonstrate the superiority of fibrin glue over mechanical stapling for mesh fixation. Thus, well-designed, randomized studies that compare glue and staple fixations for TAPP repair remain warranted. Most previous studies applied only one mesh fixation method for patients with bilateral hernias. Only one study compared glue and staple fixations in single patients with bilateral hernias [24]; interestingly, that study was not included in the review by Schäfer et al. [18].

This study protocol describes a two-by-two trial design that will be used to compare the two fixation methods in two groups of randomized patients; one group with unilateral hernias and the other group with bilateral hernias. The latter group will allow a comparison of the two methods in a single patient. This design will eliminate the inter-individual bias that may influence the unilateral hernia group. This study focuses on early postoperative pain, particularly during the first two postoperative days. In many other studies, pain is first measured one month after surgery.

### Aim of the study

The aim of this trial is to investigate early and late postoperative pain after laparoscopic hernia repair with the TAPP technique, using either fibrin glue or staples for mesh fixation.

## Methods/Design

The study was planned according to the updated Consolidated Standards of Reporting Trials (CONSORT) statement. The study design is in compliance with the Declaration of Helsinki, the Guidelines of Good Clinical Practice issued by ICH, and the requirements of Swiss regulatory authorities [25-27].

### Trial design

**Figure 1:**
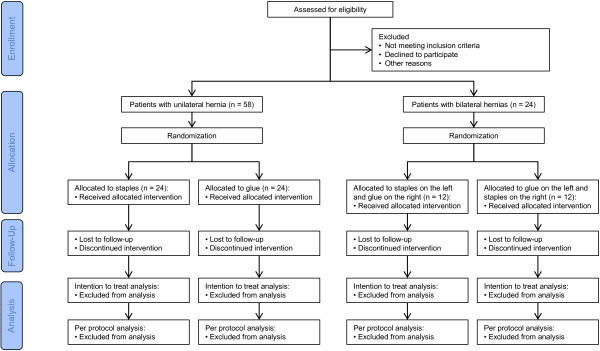
Consort diagram of the TISTA trial.

This trial is a double blind, randomized, controlled trial with a two-by-two design, conducted at a single institution. The study aims to compare pain after mesh implantation, when fixed either with staples or glue, in a superiority analysis. The CONSORT diagram of the trial is depicted in Figure 1.

### Eligibility criteria and patient recruitment

Patients scheduled for elective laparoscopic primary inguinal hernia repair at the Department of Surgery, Kantonsspital St. Gallen, will be screened for enrollment in the trial. Informed consent will be obtained, at the latest, on the day before surgery, after ascertaining that the patient meets the eligibility criteria.

Patients that match the following criteria are eligible for inclusion in the clinical trial:

• age, 18 years or older

• male gender

• primary hernia repair (no re-operations for a recurrent hernia)

• no chronic pain

• no acute pain that requires analgesics, other than paracetamol or more than a single dose of non-steroidal anti-inflammatory drug treatment within 48 h before surgery

• no current treatment with psychopharmaceutical drugs

• good compliance can be expected

• no mental incapacity

• no known incompatibility (allergies) with Tisseel compounds

• informed consent

Under the following circumstances, patients will be excluded from the trial:

• hernia is inoperable with TAPP, due to the size, previous abdominal surgery (assuming a damaged peritoneum), or expected adhesions

• repair requires an intra-operative change to another hernia repair technique (e.g. open access methods)

• the patient wishes to stop further trial participation

When the patient refuses further participation after receiving surgery, the patient will receive a safety check-up during a one-month follow-up. Otherwise, the patient will not receive the study treatment and will not require a study-related safety follow-up.

### Study objectives and endpoints

The primary objective of this trial is to show that after TAPP repair for unilateral and bilateral inguinal hernias the acute postoperative pain caused by mesh fixation with tissue glue is less than with staple fixation.

The primary efficacy endpoint of the TISTA trial is pain under physical stress at 24 h after surgery. Pain will be rated by the patient using a numeric rating scale (NRS). The NRS ranges from 0 to 10 in steps of one, with 0 representing no pain at all and 10 the worst imaginable pain. The NRS is comparable to the visual analogue scale (VAS) where the degree of pain is marked on a 10 cm long line. In practical terms, average NRS and VAS scores (in cm) can be considered equivalent. The patient is first asked to rate the pain at rest and then to rate it after physical stress. Physical stress is induced by a 90° bending of the hip joint on the operated side(s). The secondary endpoints of the trial and their definitions are summarized in Table 1.

**Table 1:** Secondary endpoints and definitions

**Secondary endpoints**	**Definitions**
Pain rated before surgery and at 4, 8, 12, 36, 48 h after surgery	Measured on a numeric rating scale from 0-10 at rest and upon bending the hip joint on the operated side
Operating time	Time, in minutes, from the first skin incision to the application of dressing
Length of hospital stay	Time, in days (with one decimal precision), from start of surgery to hospital release
Postoperative analgesic requirements	Amount (g/day) and type (paracetamol, metamizole, morphine) of analgesic required after surgery, before hospital discharge
Incidence of persistent pain (neuralgia)	Neuralgia defined as:
	• The presence of intermittent hyperesthesia, burning sensation, or jabbing pain in the ipsilateral, inguinal area nerves (genitofemoral nerve, lateral cutaneous femoral nerve, ilioinguinal and iliohypogastric nerve) [20,22,27]
	• Evaluation will be performed 14 days, 1, 3 and 12 months after surgery
	• The degree of pain will measured as described above
Postoperative morbidity	Categorized according to Dindo et al [28], and it includes:
	• Wound infection, defined as infections treated without further surgery and identified by clinical examination without microbiological confirmation
	• Hematoma or seroma formation, identified by clinical examination alone, before discharge from hospital, which does not require radiological confirmation
	• Re-operation, defined as the need for re-operation during the initial hospital stay
	• Bleeding
	• Urinary retention, urinary tract infection
	• Pulmonary infection
	• Any serious deviation from the normal postoperative course
Time to return to normal activities	Time, in days, from hospital discharge to first working day
Relapse	Within 1 year of surgery
Economic impact	Calculated based on the following factors:
	• Cost for glue, staples, and instruments to apply glue or staples
	• If relevant: cost for excess operating time for the “slower” mesh fixation
	• Length of hospital stay (cost/day)
	• Medical leave of absence (converted into a monetary amount based on an average salary)

### Standardization of treatments

Patient intra- and peri-operative care will be standardized and maintained identical for each patient, except for the mesh fixation technique. The patients will receive combined neuraxial and general anesthesia.

### Transabdominal preperitoneal patch plastic repair (TAPP)

The abdomen will be accessed and the pneumoperitoneum will be achieved with standard laparoscopic techniques. The preperitoneal space will be exposed transabdominally with a sharp incision, followed by bluntly stripping the peritoneum that overlies the inguinal anatomy. An Ultrapro® mesh (Ethicon Inc., Johnson & Johnson, Somerville, NJ, USA) will be deployed and fixed in place with either staples or glue, and then the peritoneum will be returned to its anatomical position.

### Surgical technique

At an umbilical site, a Veress needle is inserted to induce a pneumoperitoneum, and then the needle is replaced with a 10 to 12 mm optical trocar. Next, two 5 mm trocars are positioned bilaterally on the umbilical line in the iliac fossa. An incision is made in the peritoneal wall, starting at the level of the superior margin of the internal inguinal ring and at the level of the epigastric vessels. The incision is extended medially, up to the residue of the umbilical artery, and then laterally, 3 to 4 cm past the inguinal ring; the total incision length is 7 to 8 cm. In the presence of a direct hernia, the hernial sac is directly isolated and reduced. In the case of an indirect or femoral hernia, the preperitoneal parapubic adipose tissue is carefully dissected medially to expose the horizontal pubic ramus and Cooper’s ligament. Accurate dissection of the preperitoneal retrovesical tissue facilitates positioning the mesh. The internal inguinal ring is explored, and the hernial sac is isolated and reduced; this maneuver is performed to reveal the presence of perihernial lipomas, which can then be removed. Once the spermatic cord is freed from the peritoneal wall, a macroporous, partially absorbable Ultrapro mesh (Ethicon) is positioned. This type of mesh allows the surgeon to view the underlying structures, which facilitates positioning the mesh. The mesh is cut to 10 × 13 cm and placed in the preperitoneal space such that it is in medial contact with the paravesical area, it covers Cooper’s ligament, it rests on the inguinal region, and it extends laterally over the epigastric vessels.

### Stapling

For this procedure, the mesh is fixed with an Endopath Multifeed Stapler with a 10 mm shaft and helical titanium staples (Protack, Covidien, Mansfield, MA, USA). The technique that we adopted involves positioning two metal clips at the level of Cooper’s ligament and the pubic tubercle. Additionally 4 staples are placed laterally at the level of the deep inguinal ring medially and laterally to the inferior epigastric vessels with absorbable staples (Securestrap, Ethicon). The peritoneal flap is then closed with four additional absorbable staples.

### Tisseel™ tissue glue

For this procedure, the mesh is anchored with 2 mL of fibrin glue (Tisseel, Baxter, Deerfield, IL, USA). Tisseel is applied to both anterior and posterior sides of the mesh with a dedicated laparoscopic tool (Duplotip, Baxter) inserted into a 5 mm trocar. To obtain optimal bonding between the Tisseel on the mesh and the peritoneal wall, slight pressure is applied to the mesh with the Duplotip. The Tisseel is applied to the entire perimeter of the mesh, and in particular, at the level of the superior margin, the “triangle of disaster,” in proximity of the prevesical fat, to assure good adhesion. The peritoneal flap is then closed with small, continuous, absorbable 2/0 sutures.

### Postoperative care

To reduce bias, the postoperative regime is standardized for the study. All patients will receive the same intra- and perioperative care, except for the mesh fixation technique. The postoperative care will also be uniformly administered, and changes will only be made when the patient’s comfort or safety is compromised. All patients will be mobilized as soon as possible during the postoperative period. All patients will receive paracetamol (three 1 g doses per 24 h), administered either intravenously (early postoperative course) or orally (late postoperative course).

Additional analgesia will be provided as required according to the following scheme:

• First line reserve: metamizole, maximum 4 g/24 h

• Second line reserve (if necessary): morphine, 1-2 mg, delivered intravenously

The use of additional analgesics will be documented.

### Trial interventions

Patients will be randomized according to the following scheme:

Primary arm 1: Patients with a unilateral inguinal hernia

secondary arm 1: mesh fixation with tissue glue

secondary arm 2: mesh fixation with staples

Primary arm 2: Patients with bilateral inguinal hernias

secondary arm 1: right side, tissue glue; left side, staples

secondary arm 2: right side, staples; left side, tissue glue

### Methods for reducing bias

The patients will be randomly assigned to the treatment, and the patients will be blinded to the assigned treatment. In primary arm 2 (patients with bilateral hernias), the bias of individual pain perception can be eliminated, because a single individual decides (blindly) which side is more painful.

The treatment assignment will be based on a block randomized list (one for each primary arm) with variable block sizes (between 2 and 6 patients per block). After each patient signed the informed consent, his name will be entered into the study database. At the beginning of surgery, the patients scheduled for unilateral repair will be examined laparoscopically to determine whether the other side also requires repair. The final decision for uni- or bilateral repair can only be made after this intra-operative examination. Thus, randomization will occur during surgery by contacting the study nurse to receive the treatment allocation. In the absence of the study nurse, the data manager can provide the allocation information. The data manager generates the randomization list and maintains a secured copy of the assignment list. The pain scores are obtained by nurses or physicians that are not aware of the treatment assignment (observer blinding).

In the case of unforeseen postoperative complications, the treating physician (surgeon) will know the treatment assignment and can be reached by phone. Furthermore, the data manager can unblind the assignment when necessary. Any unblinding will be recorded in the study documentation.

### Sample size calculation

The sample size was calculated under the hypothesis that either glue fixation or staple fixation is superior for the primary endpoint (= pain after 24 h)(two-sided analysis). Lovisetto reported a mean VAS pain score of 1.9 for glue fixation and 2.6 for staple fixation one month after TAPP repair [19]. The observed difference of 0.7 is usually considered to be of no clinical relevance. We thus assumed a difference of 1 as a clinically relevant difference. Standard deviation was estimated to be equal 1, resulting in a standardized effect size of 1 which generally is considered clinically relevant. The sample size calculation was performed as two-sided analysis with a type I error of α = 0.05 and a power of (1-β) = 0.90.

Based on these assumptions, we calculated that 23 patients per arm would be required for unilateral hernias (t-test for two independent samples with common variance). Assuming a drop-out rate of 20%, the total number of patients needed per arm is 29, resulting in a total of 58 patients with unilateral hernias. For the second arm with bilateral hernias we calculated that 19 patients would be required (t-test for paired samples), assuming an intra-individual correlation of 0.2 (derived from a preliminary, unpublished analysis of patients with bilateral inguinal hernias that had undergone a Lichtenstein procedure). With a drop-out rate of 20%, a total of 24 patients with bilateral hernias would be required, resulting in a total of 82 patients for the entire study.

With an annual case load of approximately 100 in the previous years, we estimate that patient recruitment should be achieved within one or two years.

### Statistical analysis

The statistical analysis will be performed with SPSS Statistics for Windows (IBM Corp., Armornk, NY, USA). A two-sided p-value <0.05 will be considered statistically significant. Descriptive statistics will be used to analyze the baseline characteristics. For the superiority analysis of pain intensity at 24 h after surgery, the Mann−Whitney U and Wilcoxon tests will be applied, as appropriate. To adjust for multiple testing, the Hochberg procedure will be applied [29].

Pain measurements performed at 4, 8, 12, 24, 36, 48 h after surgery by patients with unilateral and bilateral hernias will be analyzed in one mixed model to assess the mean ranks of the NRS scores with adjustments for time, treatment, and unilateral versus bilateral hernias. To compare other continuous secondary endpoints (e.g., operating time, length of hospital stay, postoperative administered analgesics, time to return to normal activity, and economic impact), the Mann−Whitney U and Wilcoxon tests will be applied, as appropriate. Chi-square statistics will be used to compare categorical secondary endpoints (e.g., incidence of chronic pain, rate of recurrence, and postoperative morbidity). Auxiliary, non-confirmatory analyses will be performed to assess the influences of baseline patient characteristics and treatment characteristics on the primary and secondary outcomes.

Intermittent missing values will be replaced by linear interpolation (e.g. post-operative pain values within 48 h after surgery). No interim analysis is planned for this study. Data from patients withdrawn from the study will be disregarded, unless exclusion is based on a postoperative patient request, and the patient agrees to the use of existing documented data.

The confirmatory analysis will be performed based on an intention-to-treat (ITT) design, and it will adhere to ITT principles. When a protocol violation occurs, a per protocol analysis (PP) will be performed for comparison.

### Ethical and legal considerations

This study will be conducted in agreement with either the Declaration of Helsinki (Tokyo, Venice, Hong Kong, Somerset West, and Edinburgh amendments) or the local laws and regulations of the country, whichever provides the greatest protection for the patient. This protocol, the patient information sheet, and the patient consent form have been reviewed and approved by the local Ethics Committee (EKSG 12/080) and by Swissmedic (TpP_I_2012_003) prior to enrolling any patients in this trial.

All patients will be informed of the aims of the study, the possible adverse events, the procedures and possible hazards to which they will be exposed, and the mechanism of treatment allocation. They will be informed about the strict confidentiality of their patient data and told that their medical records may be reviewed for trial purposes by authorized individuals other than their treating physician. The signed consent document will be maintained by the investigator. A copy of the signed consent document will be given to the patient or the patient’s legally authorized representative.

It will be emphasized that participation is voluntary, that the patient is allowed to refuse further participation in the study at any time, and that any refusal will not influence the patient’s subsequent care. Documented informed consent must be obtained for all patients included in the study before they are registered or randomized in the study. Registration and randomization will be conducted in accordance with the national and local regulatory requirements.

Maintaining patient confidentiality is the responsibility of the investigator. During the trial, the patients will be identified solely based on an individual identification code. The trial findings will be stored in accordance with local data protection law/ICH GCP Guidelines, and they will be handled in the strictest confidence. For the protection of these data, organizational procedures have been implemented to prevent distribution of the data to unauthorized individuals.

The investigator will maintain a personal subject identification list (screening numbers with the corresponding subject names) to ensure the records can be identified when necessary.

## Discussion

This study was designed as a randomized trial with a two-by-two, parallel design. We aimed to compare patients with unilateral hernias that received mesh implants, fixed with either tissue glue or staples and to evaluate patients with bilateral hernias that received mesh implants fixed with tissue glue on one side and staples on the other side. In the latter group, the same patient will rate the pain associated with both methods; these ratings will not be influenced by inter-individual biases present in the unilateral hernia arm. To further reduce biases, both patients and observers (staff evaluating the pain) will be blinded to the assigned treatment.

We expect that mesh implants fixed with tissue glue will be associated with less postoperative pain, shorter hospital stay, and a shorter sick leave after the operation compared to implants fixed with staples. In addition to enhancing patient comfort, the tissue glue fixation will also have an economic impact, by reducing health care costs. Because we can evaluate both inter-individual and intra-individual biases in pain perception, this study will provide important insights for future studies that aim to investigate pain. This study will therefore provide novel information that is currently lacking in the literature.

### Trial status

The trial began on February 1^st^, 2013, and recruitment is ongoing.

## Abbreviations

CONSORT: Consolidated Standards of Reporting Trials; GCP: Good clinical practice; ICH: International Conference on Harmonisation; ITT: Intention to treat; NRS: Numeric rating scale (for pain measurement); PP: Per protocol; TAPP: Transabdominal preperitoneal patch plastic; TEP: Total extraperitoneal (repair); TISTA: This study’s acronym (**TI**sseel versus **STA**ples); VAS: Visual analogue scale (for pain measurement).

## Competing interests

The authors declare that they have no competing interests.

## Authors’ contributions

SAM and IT developed the original study design. SAM, UB, and IT developed the research protocol. RW and IT performed the sample size calculations. SAM, BMS, CL, and IT are responsible for the clinical input. SAM, RW, IT, and UB drafted the manuscript. All authors have approved the final manuscript.

## Pre-publication history

The pre-publication history for this paper can be accessed here:

http://www.biomedcentral.com/1471-2482/14/18/prepub
